# Full recovery of muscle function after delayed primary repair of deltoid muscle detachment

**DOI:** 10.4103/0973-6042.44144

**Published:** 2008

**Authors:** Umut Akgün, Baris Kocaoglu, Mustafa Karahan

**Affiliations:** Department of Orthopedic Surgery, Acibadem Kozyatagi Hospital, Istanbul, Turkey; 1 Department of Orthopedic Surgery, Acibadem Kadikoy Hospital, Istanbul, Turkey; 2 Department of Orthopedic Surgery, Marmara University Hospital, Istanbul, Turkey

**Keywords:** Delayed repair, deltoid, muscle, rupture

## Abstract

Detachment of the deltoid muscle and tendon is a rare complication that is reported to result in poor outcome after rotator cuff surgery. We performed a delayed primary repair of the detached deltoid in a 53-year-old female patient who underwent an open acromioplasty procedure. A successful result was achieved after surgical restoration of the deltoid muscle origin back to the acromion. At 25 months' follow-up the patient had recovered almost the full range of motion of the glenohumeral joint and was free of pain. Due to lack of literature on this rare condition, there are no well-defined treatment principles for the management of deltoid muscle detachments that develop as a complication of rotator cuff surgery. This paper describes a repair procedure for the management of deltoid muscle detachments. In addition, it discusses the importance of the guidelines that have to be followed during primary rotator cuff surgery.

## INTRODUCTION

Detachment of the deltoid muscle and tendon is a rare complication that is reported to result in poor outcome after rotator cuff surgery. There have been a few publications regarding this disorder, which may occur spontaneously or postoperatively.[1–4] The largest series reported appeared in two major articles in which the detachments were related to surgical procedures.[5] Deltoid muscle detachments after shoulder operations are reported to result in poor outcome. It is advised to perform primary repair within the first 2 weeks to achieve a good result. Reattaching the muscle has seldom been successful.[5]

In this paper, we report a case in which the deltoid muscle got detached after excessive acromioplasty. A successful result was achieved after surgical restoration of the deltoid muscle origin back to the acromion 1 year after the index operation. At 25 months' follow-up the patient had almost the full range of motion of the glenohumeral joint and was free of pain.

## CASE REPORT

A 53-year-old, right-handed woman with severe pain and loss of active abduction presented to our hospital with complaints that had started 1 year ago after an open acromioplasty procedure performed for impingement syndrome in another clinic. At that time, radiological studies had revealed an intact bony structure with a type III acromion and no tear had been noted in the rotator cuff in the MR images. A standard open anterior acromioplasty was performed under general anesthesia; the surgeon had not mentioned any perioperative complication or signs of any problem during surgery. An aggressive physical therapy protocol had been prescribed, and active and passive free range of motion was allowed immediately after surgery. A month after the operation the patient started complaining of weakness of her shoulder, especially with regard to her ability to abduct her arm. Physical therapy was continued for 3 months following surgery, after which the patient stopped attending the sessions. She consulted specialists at several different institutions and all advised conservative therapy. Conservative therapy continued unsuccessfully until the patient was referred to the senior author's institution for further evaluation.

**Figure 1:**
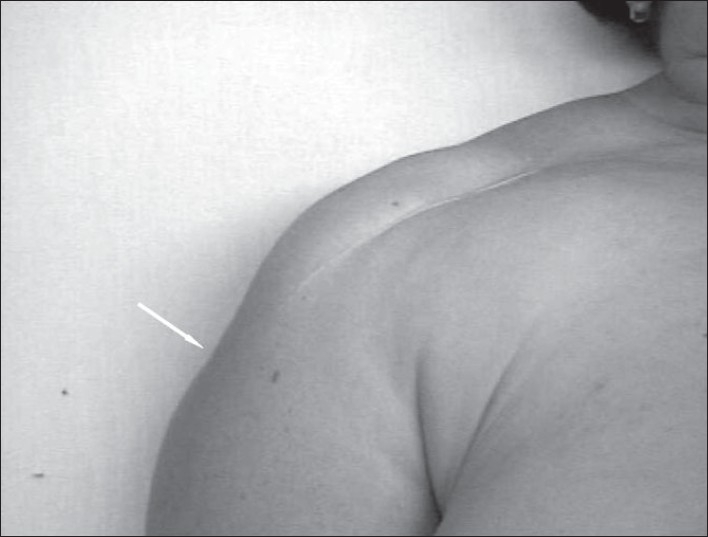
The unconventional acromioplasty open incision scar. The white arrow shows the detached deltoid insertion.

At the time of presentation to the senior author's institution, 1 year after the index operation, the patient complained of excessive pain that awakened her from her sleep and severely limited her daily activities. On physical examination of the right shoulder, an unconventional matured incision scar was visible [Figure 1]. Her right acromion was readily palpable and the bulk of the deltoid muscle could be easily palpated over the superior portion of the arm with attempts at shoulder abduction. The active range of motion (ROM) of the right shoulder was severely limited: active scapular abduction was 50° and both active external and internal rotations were limited to 10° each. Her passive scapular abduction was 120° and passive external and internal rotations were 25° and 20°, respectively. Impingement sign could not be evaluated due to the overall painful condition of the shoulder.

Based on the findings on physical examination and the history, rotator cuff tear, subacromial impingement syndrome, calcific tendonitis, axillary nerve palsy, and deltoid muscle detachment were all considered in the differential diagnoses. Careful review of the plain radiographs taken between the index and the second surgeries gave valuable information regarding the progress of the pathology. A plain anteroposterior radiograph of the right shoulder which was taken 1 month after the index surgery showed evidence of partial lateral acromionectomy, with the deltoid muscle retracted to the level of the greater tuberosity. The magnetic resonance imaging (MRI) showed that the deltoid muscle mass had retracted about 3 cm distal to the greater tuberosity which was additionally marked by an ossified tissue. We were not able to see the rotator cuff tear and the calcific tendonitis. Electromyographic (EMG) studies of the right upper extremity muscles were all normal except in the case of the deltoid muscle. The right axillary nerve conduction study showed that the distal latency was 2.8 msec (normal) and the compound muscle action potential amplitude was 4.5 mV (normal). The study was interpreted as decreased recruitment in the deltoid muscle. The diagnosis was made based on history, physical examination, the radiological studies, and the EMG study. The patient underwent a delayed primary repair of the deltoid muscle detachment, which had resulted from the excessive acromioplasty.

**Figure 2:**
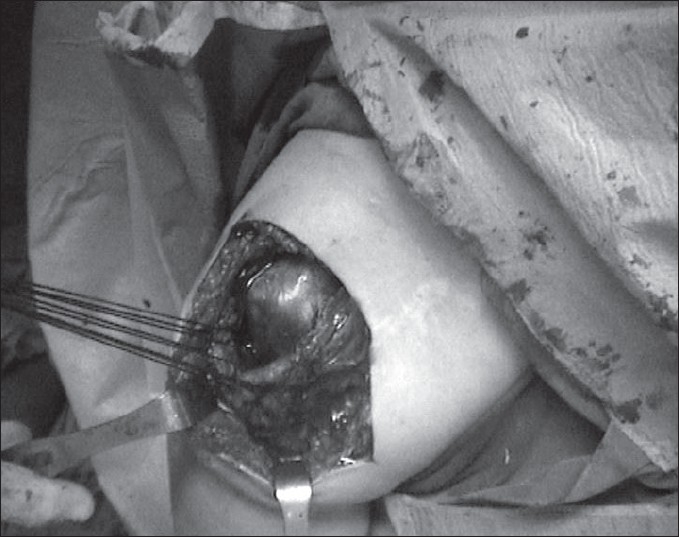
The adhesions between the proximal deltoid tendon and the proximal humerus are completely detached and the deltoid is put on a secure sling

Through a standard modified acromioplasty incision, which extended from a point over the medial and inferior part of the coracoid to a point lateral to the acromion, the skin and the subcutaneous tissues were carefully dissected. There were extensive adhesions and fibrosis of an abnormal scar tissue that did not represent muscle or tendon tissue. After debridement of the scar tissue, the acromion could be visualized. The deltoid muscle that is normally encountered at this stage of the dissection was absent. A mass of a tissue lying deep in the wound was assumed to be the deltoid muscle. Because the scar tissue had extensively covered the deltoid musculotendinous origin, this structure could not be differentiated. The dissection was extended over the acromion posteriorly until the point where healthy attachments of the deltoid muscle to the acromion were seen. The deltoid muscle tendon was then traced to exactly locate the portions detached away from the acromion. Detached parts of the tendon and the muscle mass had loosely adhered to the proximal humerus distal to the greater tuberosity. We carefully dissected away the useless scar tissue at the origin of the muscle before performing a thorough examination of the rotator cuff by rotating the humerus internally and externally until every part of the rotator cuff was visualized. The rotator cuff looked continuous and healthy. Multiple Mason-Allen sutures were passed through the deltoid muscle-tendon junction to get a good hold of the muscle [Figure 2]. While holding on to the sutures, the adhesions between the muscle and the humeral bone were separated by gentle blunt dissection. During the separation, the axillary nerve was identified and all care was taken to avoid damage to it. Ethibond sutures were then passed through the remaining acromion so that the deltoid origin would be approximated to the superior part of the acromion [Figure 3a and b]. The sutures were tied with the arm in 90° abduction.

**Figure 3a:**
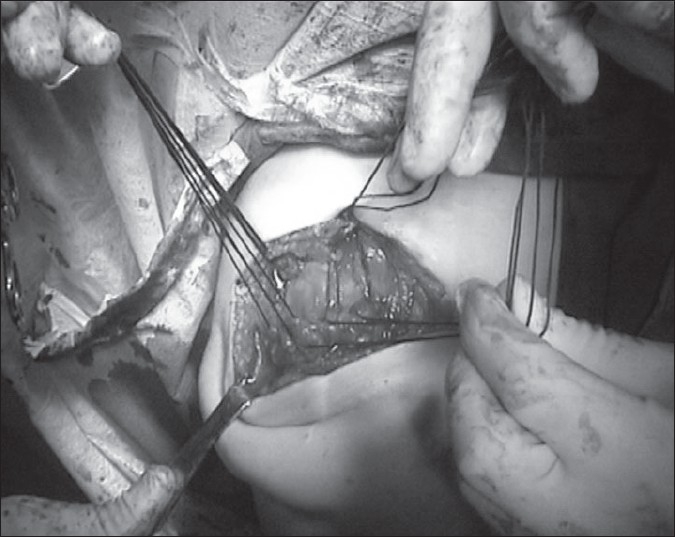
Secure fixation of the proximal deltoid to the acromion

**Figure 3b:**
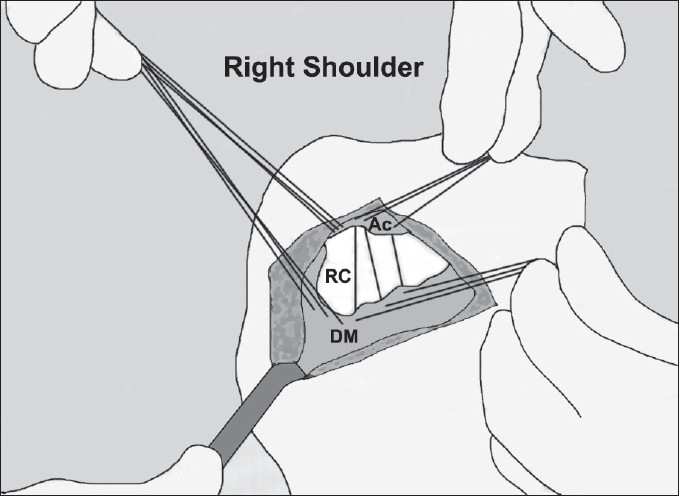
Schematic drawing of secure fixation of the proximal deltoid to the acromion. RC: rotator cuff, DM: deltoid muscle, Ac: acromion

Postoperatively, the arm was immobilized in 90° abduction for 3 weeks with a custom-made, adjustable arm holder. Starting at the third week, the arm holder was adjusted at weekly intervals, reducing the amount of abduction by 15° every week. Passive abduction was allowed for the remaining abduction range of motion. Active or passive elbow and wrist motion was allowed, starting from postoperative day 1. At 6 weeks postoperatively, aggressive physical therapy and rehabilitation was started to regain range of motion of the shoulder joint and strength of the arm muscles.

**Figure 4:**
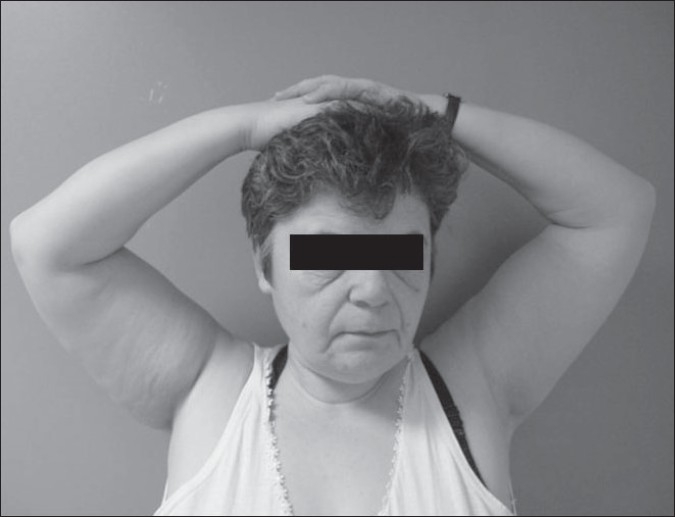
Both arms comfortably reaching the hair, representing functional external rotation and almost full scapular elevation of both arms

At the postoperative third month, the patient was able to perform her activities of daily living and by the sixth month she reported that her shoulder was almost back to normal. At the latest follow-up (25 months postoperatively), she had no functional limitation, was fully involved in her daily household activities, and could take care of her grandchildren [Figure 4]. The MR images taken 2 years after the second operation showed anatomical healing of the proximal deltoid muscle onto the acromion.

## DISCUSSION

The deltoid is the most important muscle of the shoulder. It is a multipennate muscle that arises from the anterior and superior surface of the lateral third of the clavicle (anterior deltoid), from the lateral margin and adjoining upper surface of the acromion (lateral deltoid), and from the crest of the spine of the scapula (posterior deltoid). In a normal shoulder with intact musculature, the deltoid provides 50% of the elevation power in the scapular plane.[1] Depending on the amount of damage caused, loss of deltoid function will result in limitation of shoulder activities to varying degrees. A postoperative nonfunctioning deltoid may primarily be seen in two situations: when there has been injury to the nerve supply or when there has been loss of functional continuity of the deltoid muscle.[1]

As soon as it leaves the quadrangular space, the axillary nerve splits into two major trunks. The posterior trunk gives off branches to the teres minor and posterior deltoid and terminates as the superior lateral cutaneous nerve of the arm. The anterior trunk gives off branches to the middle and then anterior deltoid, traveling 5 cm distal to the acromion. Attention should be paid to protection of the axillary nerve during surgery in this area; the incision in the deltoid-splitting approach should not extend more than 5 cm below the acromion and the shoulder should be placed in adduction and external rotation to keep the nerve away from the field. Moreover, the axillary nerve should always be identified, because the previously described safe zones are often inconsistent.[2] The approach used in exposing the pathological area, especially the type of incision used, is very important for avoiding nerve injury. In the current case, the patient had undergone an open acromioplasty in her index operation through a 10-cm-long unconventional incision perpendicular to Langer's lines, extending from the mid-clavicle to the middle deltoid laterally. The incision, by itself, indicates that the patient had undergone a nonstandard procedure. Shoulder incisions should be tailored to the shoulder pathology and detailed descriptions are given elsewhere. In the revision operation, we used an incision paralleling Langer's lines, extending from the posterior part of the deltoid insertion on the acromion anteriorly to the lateral part of the coracoid process.

The extensive acromionectomy performed by the index surgeon in this patient was an unintentional procedure. Lateral acromionectomy has long been abandoned because, although it provides excellent exposure, the deltoid origin is weakened.[3] Acromial morphology should always be respected and only the necessary amount of bone should be resected for exposure or treatment. Lateral acromionectomy leads to a poor result and should not be performed. In cases where acromioplasty is indicated for treatment purposes, aggressive acromioplasty should be avoided because it may result in excessive removal of the acromion and the deltoid attachment sites may thereby be jeopardized. The principles of adequate acromioplasty are well outlined in the literature.[5]

Deltoid detachment was the cause of treatment failure in this patient who was operated for subacromial impingement syndrome. With recent advances, most symptomatic disorders of the rotator cuff and the acromion can be treated successfully, allowing return to premorbid levels of activity. Unsuccessful results after operative treatment have been reported in patients who have any of the following: a large or massive tear; a rotator cuff tendon of poor quality; an inadequate repair due to insufficient mobilization of the rotator cuff at the time of the procedure; damage to the deltoid origin, with or without lateral or radical acromionectomy, at the time of the procedure; removal of an insufficient amount of the anterior portion of the acromion; inadequate external support postoperatively; and improper rehabilitation.[5] Of these, deltoid detachment results in the worst outcome.

The three parts of the deltoid muscle must be evaluated separately as far as muscle action is concerned. The lateral deltoid when acting alone is a powerful abductor of the arm in the plane of the scapula. Loss of function of the posterior deltoid is not a significant problem since the latissimus dorsi muscle will act as the strong synergistic muscle. The anterior deltoid flexes and internally rotates the shoulder. There is no replacement for this powerful muscle. The situation can be especially bad if more than one portion of the deltoid is involved in the pathology. In the current case, the lateral and the anterior part of the deltoid was avulsed from the acromion. It is not possible to say when exactly the detachment took place. The index surgeon has stated that nothing extraordinary took place during surgery but plain radiographs taken the day after surgery show that lateral acromionectomy had been performed, and the contour of the deltoid muscle is drawn away from the acromion. We believe that some detachment took place during surgery and was later aggravated by the aggressive postoperative protocol. The deltoid detachment, in the first place, could have been avoided had a standard anterior acromioplasty been performed. When noticed, any part of the deltoid muscle should be repaired and re-attached to the bone and deltotrapezial fasia with strong sutures.

Postoperative MR images taken after the second surgery showed that the deltoid muscle was attached to the acromion successfully. The images are proof that even a delayed repair of the deltoid muscle detachment may result in a favorable outcome. This case is a good example to prove that complicated cases can be diagnosed with a careful physical examination and simple diagnostic tools such as plain radiography and EMG study. Preoperatively, MR imaging was not requested because the final diagnosis was already made when plain radiography revealed the contour of the muscle mass lying on the proximal humeral shaft and the extent of lateral acromionectomy. Decreased recruitment of the deltoid muscle on the EMG study further confirmed that the symptoms were due to a functional discontinuity in the muscle rather than a neurological disorder.

We achieved a very good result by repairing the deltoid muscle back to the acromion even after 1 year of detachment. Review of the literature strongly suggests that surgical treatment of deltoid detachment has seldom been successful.[1,3–5] We believe that if there is sufficient amount of bone left in the acromion and if there is EMG activity in the muscle, an attempt should be made to reattach the deltoid to the bone and be followed by a careful and slow postoperative rehabilitation protocol.

Delayed primary repair of the detached deltoid was performed in the current case. Due to lack of literature for this rare condition, there are no well-defined treatment principles for the management of deltoid muscle detachments that develop as a complication of rotator cuff surgery. This paper describes a repair procedure in the management of deltoid muscle detachments. In addition, it discusses the importance of guidelines that have to be followed during primary rotator cuff surgery.
